# Is Hearing Impairment Causally Associated With Falls? Evidence From a Two-Sample Mendelian Randomization Study

**DOI:** 10.3389/fneur.2022.876165

**Published:** 2022-04-25

**Authors:** Jun Wang, Dan Liu, E. Tian, Zhao-Qi Guo, Jing-Yu Chen, Wei-Jia Kong, Su-Lin Zhang

**Affiliations:** ^1^Department of Otorhinolaryngology, Union Hospital, Tongji Medical College, Huazhong University of Science and Technology, Wuhan, China; ^2^Union Hospital, Institute of Otorhinolaryngology, Tongji Medical College, Huazhong University of Science and Technology, Wuhan, China

**Keywords:** hearing impairment, falls, Mendelian randomization, causal association, genome-wide association study

## Abstract

**Background:**

Observational studies have suggested that hearing impairment (HI) was associated with the risk of falls, but it remains unclear if this association is of causal nature.

**Methods:**

A two-sample Mendelian randomization (MR) study was conducted to investigate the causal association between HI and falls in individuals of European descent. Summary data on the association of single nucleotide polymorphisms (SNPs) with HI were obtained from the hitherto largest genome-wide association study (GWAS) (*n* = 323,978), and statistics on the association of SNPs with falls were extracted from another recently published GWAS (*n* = 461,725). MR Steiger filtering method was applied to determine the causal direction between HI and falls. Inverse-variance weighted (IVW) method was employed as the main approach to analyze the causal association between HI and falls, whereas weighted median, simple mode, weighted mode, and MR-Egger methods were used as complementary analyses. The MR-Egger intercept test, the MR-PRESSO test, and Cochran's Q statistic were performed to detect the potential directional pleiotropy and heterogeneity, respectively. The odds ratio (OR) with 95% confidence intervals (CIs) was used to evaluate this association.

**Results:**

A total of 18 SNPs were identified as valid instrumental variables in our two-sample MR analysis. The positive causality between HI and risk of falls was indicated by IVW [OR 1.108 (95% CI 1.028, 1.194), *p* = 0.007]. The sensitivity analyses yielded comparable results. The “leave-one-out” analysis proved that lack of a single SNP did not affect the robustness of our results. The MR-Egger intercept test exhibited that genetic pleiotropy did not bias the results [intercept = −2.4E−04, SE = 0.001, *p* = 0.832]. Cochran's Q test revealed no heterogeneity.

**Conclusion:**

Our MR study revealed a causal association between genetically predicted HI and falls. These results provide further evidence supporting the need to effectively manage HI to minimize fall risks and improve quality of life.

## Introduction

Falls have become a major public healthcare problem in many countries (1). The rate of falls varies with socioeconomic status and increases with age. It is estimated that roughly 30–40% of adults over 65 years suffer from fall each year across the globe, and approximately half of them suffer from recurrent falls (2–4). Falls can result in such consequences as handicaps, depression, loss of independence, fear of falling, functional impairment, and even deaths and can tend to pose a substantial economic burden on the victims and the society at large (1). Previous studies focused on contributors to falling, such as environmental factors (5) (e.g., poor lighting and surface irregularities) and intrinsic factors (6, 7) (e.g., balance disorders, vitamin D deficiency, cognitive and sensory impairment, diabetes, and depression). Further understanding of the risk factors is warranted to better prevent and manage falls.

Hearing impairment (HI) represents one of the most common sensory dysfunctions (8, 9), which pose a great burden on healthcare resources. HI is characterized by a slow onset and progressive deterioration and tends to go unrecognized and under-treated (10). Evidence from epidemiological studies indicated that HI increases the falls risk (6, 11, 12), but the association remains controversial due to reverse causality and confounding effects (6, 13), which render the interpretation of these findings difficult and their implication uncertain (14). A randomized controlled trial (RCT) is the best approach to demonstrate the association between HI and fall (15). However, RCTs are not always feasible due to the complexity of study design, financial or ethical constraints, and/or difficulties involved in the collection of a large sample (16, 17). Thus, Mendelian randomization (MR) can effectively remedy the shortcomings of classical observational studies and offers an effective methodology to examine the etiology of a condition (18, 19).

The MR is an approach that employs single nucleotide polymorphisms (SNPs) as instrumental variables (IVs) of the exposure to assess the causal effects of the exposure on an outcome (19). In comparison to traditional epidemiological research, the MR approach draws on Mendel's laws of segregation and independent assortment (20), by which MR can avoid biased associations coming from interfering or reverse causal effects (21). The principal advantages of the one-sample MR approach over other alternatives are the flexibility to conduct rigorous MR, and the capability of evaluating the independence and exclusion restriction assumptions by assessing confounders at the individual level (22). Nonetheless, the limitations may affect the estimation of causality in one-sample MR datasets (e.g., traditionally low power, selection bias, weak instrument bias, and winner's curse) (23, 24). Different from the one-sample MR, its two-sample counterpart is able to assess causal relationships between a variety of exposures and outcomes, which might not be achieved with a single sample technique (25). Additionally, it increases sample size and enhances the power of MR analyses, with plenty of data on exposures and outcomes that can be used for interrogation, and these may not be feasible or affordable in the measurement of the same set of individuals (22). Thus, in this study, we used a two-sample MR approach to investigate whether the observational associations between HI and falls are likely causal, and the directionality of their relationship.

## Materials and Methods

### Study Design

**Figure 1:**
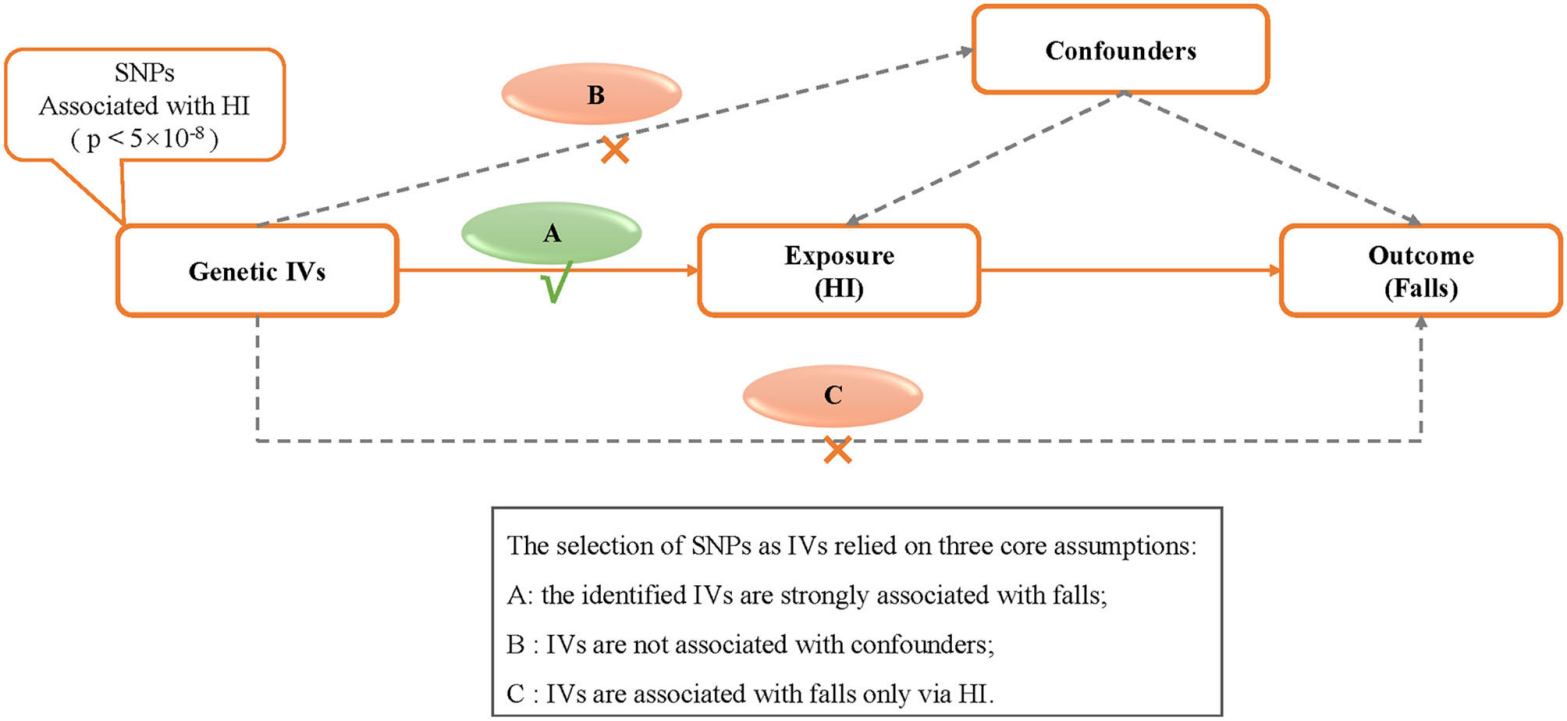
Design of the two-sample Mendelian randomization study. IVs, instrumental variables; HI, hearing impairment; SNP, single nucleotide polymorphism.

The MR study relied on three core assumptions (21): (1) the identified IVs are strongly associated with falls; (2) IVs are not associated with confounders; and (3) IVs are associated with falls only *via* HI. The MR schematic is shown in Figure 1.

### Data Sources

The summary data showing the associations between SNPs and HI were from the UK Biobank. The UK Biobank is a national health research resource involving 502,639 European participants aged 37–70 years, recruited from across the UK between 2006 and 2010. In the present study, we utilized publicly accessible datasets from published studies in which formal consent from participants and ethical approval by relevant committees had been obtained. Thus, no additional ethics approval was required. The dataset from the UK Biobank was made available to the research communities through the genome-wide association study (GWAS) database, which is a database regarding the genetic associations from large population-based cohorts of Europeans in the OpenGWAS database (26). The characteristics of the exposure and outcome of the GWAS data are detailed in Supplementary Table S1 in the supplementary material.

### HI GWAS Dataset

For the exposure data set of HI, we obtained the analysis results of GWAS that involves 323,978 individuals of European ancestry (84,839 cases and 239,139 controls) to generate the IVs (https://gwas.mrcieu.ac.uk/datasets/ukb-a-257/). Participants were assigned case/control status based on their responses to questionnaire measures regarding hearing difficulty. Self-reported hearing status data were based on the responses to the question: “Do you have any difficulty with your hearing”? Subjects who responded “Yes” were coded as having a hearing impairment, whereas “No” signified not having a hearing impairment.

### Fall GWAS Dataset

As for the outcome datasets of the GWAS, data on falls were taken from another independent GWAS analysis that includes 461,725 individuals of European ancestry (89,076 cases and 372,649 controls) (https://gwas.mrcieu.ac.uk/datasets/ukb-b-2,535/). The falls dataset of UK Biobank was self-reported by participants *via* a touch screen questionnaire. Fall cases were defined as subjects who responded “Yes” to the question “In the last year have you had fallen down for any reason (i.e., various extrinsic and intrinsic factors predisposing adults to fall)?” (27).

### SNP Selection

First, to ascertain the association with HI as IVs, we selected independent genetic variants with genome-wide significance (*p* < 5 × 10^−8^) are selected as the potential instruments from the corresponding datasets. Then, to avoid bias due to linkage disequilibrium (LD) relationship in the analysis (28, 29), the LD of SNPs closely associated with HI had to satisfy the following conditions, i.e., *r*^2^ < 0.001 and distance > 10,000 kb (28). Palindromic SNPs with intermediate allele frequencies were excluded from the selected instrumental SNPs (palindromic SNPs refer to SNPs with the A/T or G/C alleles and “intermediate allele frequencies” refer to 0.01 < allele frequency <0.30). SNPs with the wrong causal direction identified by the MR Steiger filter are excluded. SNPs with a minor allele frequency (MAF) of < 0.01 were also excluded to avoid potential statistical bias resulting from the original GWAS, since they usually carry low confidence. Additionally, to rule out the influence of known confounders on the causality assessment, potential secondary phenotypes of the selected SNPs were manually browsed with the PhenoScanner (http://www.phenoscanner.medschl.cam.ac.uk). Finally, we calculated the *F-*statistics for the SNPs to measure the strength of the instruments (30). IVs with an *F*-statistic less than 10 were excluded and are generally regarded as a “weak instrument” (30).

### MR Analysis

To perform the data analysis, individual estimates of the causal effect of exposure on site-specific outcomes mediated by each instrumental SNP were computed as the Wald ratio (31). We calculated the strength of the association between HI and falls by using the inverse-variance weighted (IVW) method as the main analysis and the MR-Egger, weighted median, simple mode, and weight mode methods as complementary analyses (32). The causal effects were measured in the odds ratio (OR). Then, Cochran's Q statistic (33, 34) was employed to estimate heterogeneity from each SNP. The MR-Egger intercept test and the MR-PRESSO test were utilized to evaluate the bias stemming from ineffective IVs and the potential horizontal pleiotropy (35–37). In addition, we performed “leave-one-out” sensitivity analysis to determine whether the result was affected by a single SNP (36). We applied the R package “TwoSampleMR” by following the guidelines from the developers (https://mrcieu.github.io/TwoSampleMR). All analyses were conducted using R software (version 4.1.1, the R Foundation for Statistical Computing, Vienna, Austria). A two-tailed *p*-value of less than 0.05 was considered statistically significant.

## Results

### Genetic Variants Selection

In total, 23 SNPs were successfully extracted from the HI GWAS dataset (*p* <5 × 10^−8^). However, 4 SNPs (rs1126809, rs13277721, rs34656207, and rs9296413) were removed because of their possible associations with confounding traits. To be exact, rs1126809 was associated with vitiligo and carcinoma; rs13277721 was associated with mood swings; rs34656207 was associated with rheumatoid arthritis, ankylosing spondylitis, diabetes, and disability or infirmity; and rs9296413 was associated with hypertension (Supplementary Table S2). Moreover, one SNP(rs12660376) was dropped due to the dataset had no corresponding effector gene. After exclusion of these 5 SNPs, our two-sample analysis identified the remaining eighteen SNPs as IVs. No LD was found among these SNPs, and the phenotype variance explained by genetics was 0.25%. The *F*-statistic of these SNPs ranged from 30 to 97 (general *F*-statistic = 45), suggesting that they satisfied the strong relevance assumption of MR and that “weak instrument” bias was unlikely. A total of 18 SNPs included in our analysis are shown in Table 1.

**Table 1:** The characteristics of eighteen SNPs and their genetic associations with HI and falls.

**SNP**	**Gene**	**Chr**	**EA**	**OA**	**EAF**	***F*** **-statistics**	**SNP-HI association**			**SNP-Falls association**		
							**Beta**	**SE**	***p*** **-value**	**Beta**	**SE**	***p*** **-value**
rs10901863	CTBP2	10	T	C	0.2676	67	0.0103	0.0012	2.99E-16	3.55E-07	0.0014	1.00
rs11238325	GRB10	7	T	C	0.7337	30	0.0067	0.0012	4.50E-08	0.0006	0.0013	0.67
rs11881070	TMPRSS9	19	T	C	0.2890	39	−0.0075	0.0012	3.67E-10	1.83E-05	0.0013	0.99
rs13147559	CLRN2	4	G	C	0.1332	30	0.0088	0.0016	4.36E-08	0.0012	0.0017	0.48
rs13172686	ARHGEF28	5	C	T	0.4708	82	0.0099	0.0011	1.25E-19	−0.0015	0.0012	0.20
rs1566129	NID2	14	C	T	0.5858	33	−0.0064	0.0011	7.44E-09	0.0004	0.0012	0.77
rs36062310	KLHDC7B	22	A	G	0.0433	97	0.0262	0.0027	8.50E-23	0.0053	0.0029	0.07
rs4732339	TMEM213	7	A	G	0.5849	30	0.0060	0.0011	4.05E-08	−6.02E-06	0.0012	1.00
rs4859223	TMEM207	3	A	T	0.3311	40	−0.0073	0.0012	3.08E-10	−0.0018	0.0013	0.15
rs55635402	TUB	11	G	A	0.1959	40	−0.0086	0.0014	3.02E-10	−0.0014	0.0015	0.34
rs5756799	TRIOBP	22	T	G	0.4602	40	0.0069	0.0011	2.80E-10	0.0001	0.0012	0.91
rs67307131	PHLDB1	11	C	T	0.3468	51	0.0081	0.0011	9.98E-13	0.0012	0.0012	0.34
rs6902016	SYNJ2	6	T	C	0.5134	51	0.0077	0.0011	1.11E-12	0.0011	0.0012	0.34
rs72930982	CCDC68	18	G	A	0.2148	30	0.0073	0.0013	3.49E-08	0.0020	0.0014	0.16
rs741475	AC007879.2	2	T	C	0.5776	35	−0.0065	0.0011	4.09E-09	−0.0018	0.0012	0.13
rs7525101	LMX1A	1	T	C	0.4398	35	0.0064	0.0011	3.65E-09	0.0007	0.0012	0.58
rs78417468	MMP2	16	A	G	0.2255	37	−0.0079	0.0013	1.22E-09	−0.0012	0.0014	0.37
rs9493627	EYA4	6	A	G	0.3200	45	0.0078	0.0012	1.95E-11	0.0021	0.0013	0.10

*SNP, single nucleotide polymorphism; HI, hearing impairment; Chr, chromosome; EA, effect allele; OA, other allele; EAF, frequency of effect allele; SE, standard error*.

### MR and Sensitivity Analyses

**Figure 2:**
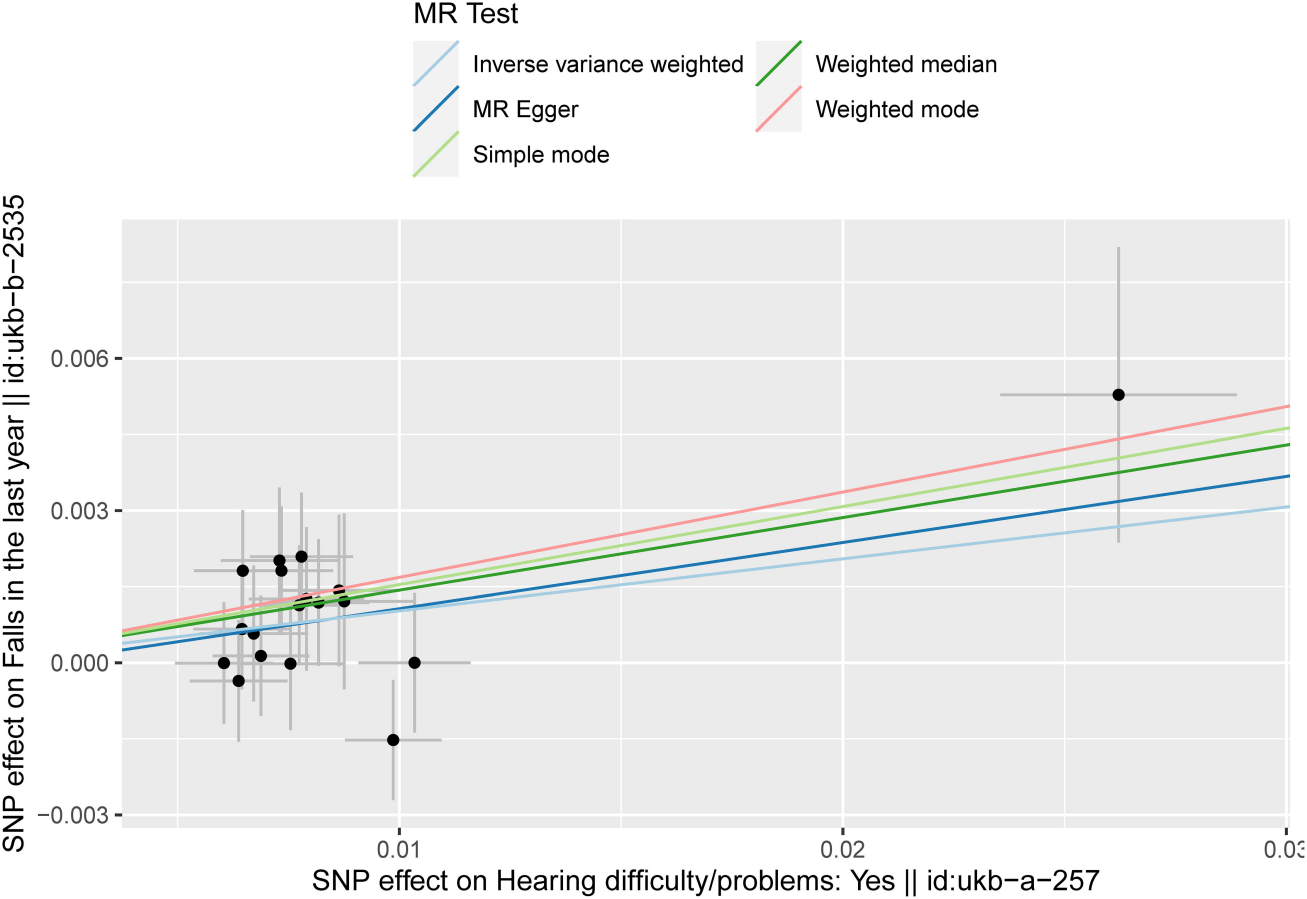
Scatter plot of the effects of genetic variants on the HI and falls. The slopes of the solid lines denote the magnitudes of the associations estimated from the MR analyses. HI, hearing impairment; MR, Mendelian randomization; SNP, single-nucleotide polymorphism.

**Figure 3:**
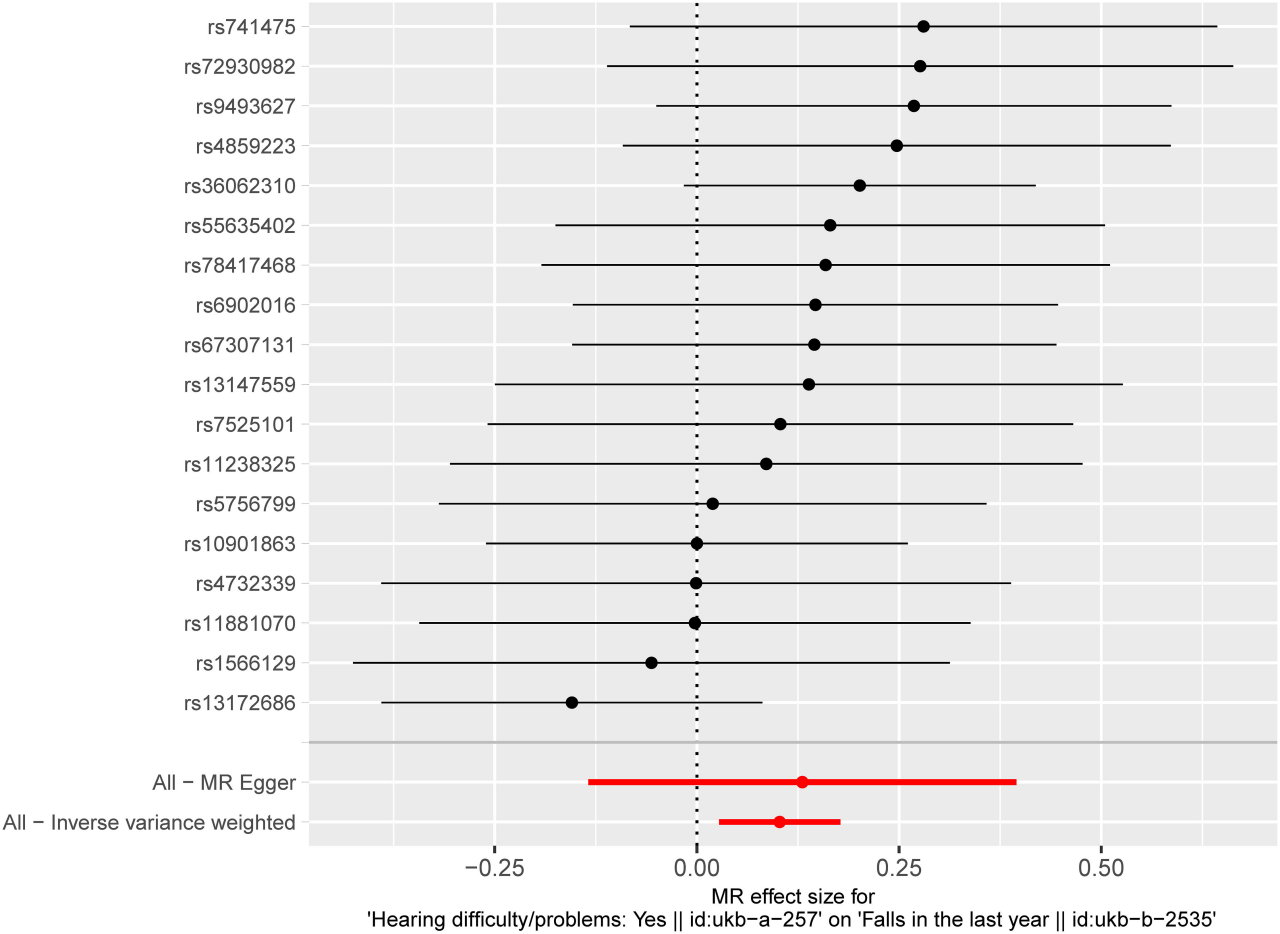
Fixed-effect IVW analysis of the causal association of HI with falls. The black dots and bars indicate the causal estimate and 95% CI using each SNP. The red dot and bar indicate the overall estimate and 95% CI meta-analyzed by fixed-effect IVW method. IVW, inverse-variance weighted; HI, hearing impairment; CI, confidence interval; SNP, single nucleotide polymorphism.

**Figure 4:**
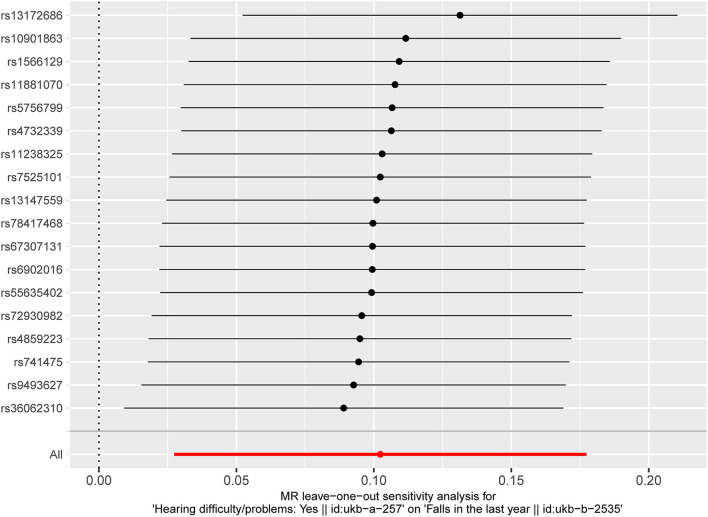
“Leave-one-out” analysis of the causal association of HI with falls. The black dots and bars indicate the causal estimate and 95% CI when an SNP was removed in turn. The red dot and bar indicate the overall estimate and 95% CI using the fixed-effect IVW method. HI, hearing impairment; CI, confidence interval; SNP, single nucleotide polymorphism; IVW, inverse-variance weighted.

The results of fixed-effect IVW estimates showed that HI was significantly associated with a higher risk of falls [OR 1.108 (95% CI 1.028, 1.194), *p* = 0.007] (Table 2 and Figures 2, 3). Cochran's Q statistic for the IVW method was 11.40 (*p* = 0.835), indicating the heterogeneity was low and the reliability for the causal effect was relatively high. Moreover, weighted median [OR 1.154 (95% CI 1.039, 1.282), *p* = 0.008], simple mode [OR 1.167 (95% CI 0.959, 1.419), *p* = 0.141], weighted mode [OR 1.183 (95% CI 0.975, 1.436), *p* = 0.107], and MR-Egger [OR 1.139 (95% CI 0.874, 1.484), *p* = 0.349] also yielded consistent results (Table 2 and Figure 2). The “leave-one-out” analysis indicated that the MR analysis was reliable of our results (Figure 4 and Supplementary Table S3). MR-Egger regression was used to assess the horizontal pleiotropy between IVs and outcome, and our results indicated no evidence for a significant intercept [intercept = −2.4E−04, SE = 0.001, *p* = 0.832] (Table 3). MR-PRESSO results also showed that no horizontal pleiotropy in our study (*p* = 0.835). The funnel plot showed general symmetry (Supplementary Figure S1), suggesting that there was no heterogeneity or horizontal pleiotropy. In addition, there was a significant correlation between HI and falls in MR analysis that included five SNPs dropped due to potential pleiotropy (Supplementary Table S4). Therefore, our results supported that there exists a causal association between HI and falls.

**Table 2:** Association of HI with falls risk using various methods.

**Methods**	**OR**	**LCI**	**UCI**	***p*** **-value**
Fixed-effect IVW	1.108	1.028	1.194	0.007**
Weighted median	1.154	1.039	1.282	0.008**
Simple mode	1.167	0.959	1.419	0.141
Weighted mode	1.183	0.975	1.436	0.107
MR-Egger	1.139	0.874	1.484	0.349

** *p < 0.01*.

*HI, hearing impairment; OR, odds ratio; LCI, lower confidence interval; UCI, upper confidence interval; IVW, inverse-variance weighted*.

**Table 3:** MR-Egger intercept test results of the association between HI and falls.

**Estimates**	**SE**	**LCI**	**UCI**	***p*** **-value**
−2.4e −04	0.001	−0.002	0.002	0.832

*MR, Mendelian randomization; HI, hearing impairment; LCI, lower confidence interval; UCI, upper confidence interval*.

## Discussion

We, for the first time, employed a two-sample MR study to examine the causal relationships between HI and falls on the basis of the summary level data of large GWASs. Our analysis provided evidence of the causal links between genetically predicted HI and falls. Consistent estimates were observed in sensitivity analyses, suggesting the association was robust and the horizontal pleiotropy was minimal.

Our results suggested that the genetic liability to HI exerts an independent effect on falls. Although the association between HI and falls has been reported previously in observational studies (11, 12, 38–40), this association observed in uncontrolled studies has been controversial (6, 13). Heitz et al. (5) found that self-reported hearing loss was significantly associated with the increased risk of falls, but the relationship weakened with adjustment for cardiovascular, vision, and emotional factors and disappeared when controlling for vestibular vertigo. Lopez et al. (11), in a longitudinal study, found that self-reported HI was significantly associated with the increased risks of suffering a fall, but not with injuries from a fall. Thus, due to the difficulty in observational epidemiological studies to eliminate the bias (e.g., reverse causality and confounding effects), etiological interpretation might have some limitations (14, 16).

In the present study, we selected SNPs with the genome-wide association and independent inheritance as IVs from HI GWAS dataset to detect their causal association with falls. To make our conclusions more reliable, we employed a range of well-established sensitivity methods to control for pleiotropy and heterogeneity and to ensure consistency of results. In MR, we minimized the confounding factors by applying a random combination of alleles against Mendel's second law. Additionally, reverse causality was also ruled out since genetic variants were fixed at conception and could not be altered by disease processes. As a result, our evidence had a high-level precision and stability.

Maintaining balance in the upright stance is maintained by the integrated input of vision, vestibular and somatic sensation into the central nervous system, resulting in a context-specific motor response *via* static and dynamic posture modifications (41, 42). Multiple theories were proposed to explain the association between HI and falls. The first hypothesis is the coexistent vestibular pathology: the peripheral vestibular organs, which collect information on the physical position, movement, and balance, are located in the inner ear, close to the auditory organs (43). Thus, HI is often concomitant with vestibular dysfunction and balance difficulties (44). The second assumption is the cognitive load hypothesis, which postulates that HI may increase cognitive load, thereby reducing the cognitive capacity remaining for balance, especially during walking (45, 46). The third hypothesis believes that individuals with sensory dysfunction may have reduced auditory and spatial awareness of their immediate surroundings, rendering them more likely to suffer from accidents and accidental injuries (47). The fourth theory is based on the effect of multisensory integration, i.e., the auditory system is a perceptual system that engages in the perception of the dynamic environment and in complex representations of 3D space through vision and touch (48). Thus, the abnormality in the integration of different sensations or modalities might lead to falls when relevant factors of balance are significantly altered (49).

Additionally, HI is a condition among older adults and can be treated with hearing amplification to improve balance ability. Lacerda et al. (50), in a prospective clinical study, employed an SF-36 questionnaire to examine the effects of bilateral hearing aid on the quality of life and found that the quality of life was improved and the fear-of-falling reduced 4 months after fitting. Parietti-Winkler et al. (48) also evaluated the effect of unilateral cochlear implantation on the modalities of balance control and sensorimotor and revealed that the balance performance of cochlear implantees reached a near-normal level compared to the age-matched healthy controls. These findings confirmed that the restoration of the ability to gather auditory information might contribute to improved balance regulation in patients with amplified hearing. Thus, these findings further underscore the importance of hearing healthcare.

The present study has the following strengths. First, this is the first two-sample MR study to confirm the causal association between HI and falls by using the summary level data of large GWASs. Second, a series of sensitivity analyses were conducted to further verify the hypothesis, making our findings more reliable. However, some limitations of our MR analysis need to be mentioned. First, the participants in the HI GWAS dataset might have overlapped with the participants in the fall GWAS dataset. In this study, we were unable to estimate the degree of overlap among participants, which might lead to weak instrument bias (51). Thus, we conducted an analysis to calculate the lower limit of a one-sided 95% CI for the *F*-statistic, where the result was 37.872 and considerable weak instrument bias would not be expected (51). Second, the summary of GWAS data merely concerned individuals of European descent, and our results might not be fully representative of the whole population. Therefore, care should be exercised to extrapolate our conclusion to other racial and ethnic populations. Third, the two-sample MR study only provides an estimate of the putative causal effect, and further studies are required to estimate the direct causal effect of HI upon falls.

## Conclusion

Our findings indicated that there was a causal association between HI and falls. Since individuals with HI are potentially at risk of falls even without vestibular disease or balance impairment, our findings suggested that sufficient attention should be paid to HI at every single link of hearing screening, diagnosis, treatment, and prognosis evaluation. Our study provided further evidence that supports the need to effectively manage the HI to minimize fall risks and improve quality of life.

## Data Availability Statement

Publicly available datasets were analyzed in this study. This data can be found here: Medical Research Council (MRC) Integrative Epidemiology Unit (IEU) OpenGWAS project, https://gwas.mrcieu.ac.uk/datasets/ukb-a-257/ and https://gwas.mrcieu.ac.uk/datasets/ukb-b-2535/.

## Ethics Statement

Ethical review and approval was not required for the study on human participants in accordance with the local legislation and institutional requirements. Written informed consent from the patients/participants or patients/participants' legal guardian/next of kin was not required to participate in this study in accordance with the national legislation and the institutional requirements.

## Author Contributions

S-LZ, W-JK, and JW conceptualized and designed the study. JW, DL, and Z-QG prepared and analyzed the data and drafted the manuscript. All co-authors contributed to the manuscript's modifications and approved the final version. All authors contributed to the article and approved the submitted version.

## Funding

This work was supported by grants from the National Natural Science Foundation of China (Nos. 82171152 and 81873701) and the National Twelfth Five-Year Research Program of China (No. 2012BAI12B02).

## Conflict of Interest

The authors declare that the research was conducted in the absence of any commercial or financial relationships that could be construed as a potential conflict of interest.

## Publisher's Note

All claims expressed in this article are solely those of the authors and do not necessarily represent those of their affiliated organizations, or those of the publisher, the editors and the reviewers. Any product that may be evaluated in this article, or claim that may be made by its manufacturer, is not guaranteed or endorsed by the publisher.

## Abbreviations

HI: hearing impairment
MR: Mendelian randomization
SNP: single nucleotide polymorphism
GWAS: genome-wide association study
IVW: inverse-variance weighting
OR: odds ratio
CI: confidence interval
IV: instrumental variable
RCT: randomized controlled trial.
