# Supplementary material for: Is Hearing Impairment Causally Associated With Falls? Evidence From a Two-Sample Mendelian Randomization Study
Source: Front Neurol. 2022 Apr 25;13:876165. doi: 10.3389/fneur.2022.876165 (PMC9082948; doi:10.3389/fneur.2022.876165)
Supplement: Supplementary file 1 [file Data_Sheet_1.docx]

**SUPPLEMENTAL MATERIAL**

**Supplementary methods**

***Self-reported*** ***hearing impairment*** ***(HI)***

Pure tone audiometry, the gold standard of hearing function, requires an audiologist, a quiet environment, and significantly long time and therefore poses challenge on the collection of the large samples needed for large GWASs. Evidence on the validity of self-reported HI suggests that self-reporting is appropriate for the evaluation of hearing loss when no other objective measurements are available [1,2], and subjective and objective measurements may well be associated, in many ways, with functional outcomes, such as HI [3]. Therefore, self-reported HI may be a suitable metric for large epidemiological studies [4-6].

**References:**

1. Gomez MI, Hwang SA, Sobotova L, et al. A comparison of self-reported hearing loss and audiometry in a cohort of New York farmers. *J Speech Lang Hear Res* (2001)44(6):1201-1208.
2. Hickson, L, Lind, C, et al. Hearing and vision in healthy older Australians: objective and self-report measures. *Advances in Speech Language Pathology* (1999) 1(2), 95-105.
3. Choi JS, Betz J, Deal J, et al. A comparison of self-report and audiometric measures of hearing and their associations with functional outcomes in older adults. J Aging Health (2016) 28(5):890-910.
4. Valete-Rosalino CM, Rozenfeld S. Auditory screening in the elderly: comparison between self-report and audiometry. *Braz J Otorhinolaryngol* (2005) 71(2):193-200.

5. Deepthi R, Kasthuri A. Validation of the use of self-reported hearing loss and the Hearing Handicap Inventory for elderly among rural Indian elderly population. *Arch Gerontol Geriatr* (2012) 55(3):762-767.

6. Diao M, Sun J, Jiang T, Tian F, Jia Z, Liu Y *et al*. Comparison between self-reported hearing and measured hearing thresholds of the elderly in China. *Ear Hear* (2014) 35(5):e228-e232.

***F*-statistic**

We computed the *F*-statistic of each SNP using the following formula [7,8]: *F*-statistic = (β/SE)^2^, where Beta is the per allele effect size of the association between each SNP and phenotype, SE is the standard error.

**References:**

7. Chen L, Yang H, Li H, He C, Yang L, Lv G. Insights into modifiable risk factors of cholelithiasis: A Mendelian randomization study [published online ahead of print, 2021 Oct 8]. Hepatology. 2021;10.1002/hep.32183.

8. Jiang J, Shao M, Wu X. Vitamin D and risk of ankylosing spondylitis: A two-sample mendelian randomization study. Hum Immunol. 2022;83(1):81-85.

**Supplemental tables**

**Table S1.** Details of datasets used for analyses.

| **Exposure/Outcome** | **Consortium** | **Participants** | **Web source if publicly available** |
| --- | --- | --- | --- |
| Hearing impairment | MRC-IUE | 323,978 individuals (84,839 hearing impairment cases and 239,139 controls) of European ancestry | <https://gwas.mrcieu.ac.uk/datasets/ukb-a-257/> |
| Falls | Neale Lab | 461,725 individuals (89,076 fall cases and 372,649 controls) of European ancestry | <https://gwas.mrcieu.ac.uk/datasets/ukb-a-262/> |

**Table S2.** Association of 4 SNPs with confounding traits at a genome-wide significance level (P < 5×10^-8^).

| **SNP** | **Gene** | **Chr** | **EA** | **OA** | **EAF** | **Confounding traits** |
| --- | --- | --- | --- | --- | --- | --- |
| rs1126809 | TYR | 11 | A | G | 0.3048 | Vitiligo, Carcinoma |
| rs13277721 | AGO2 | 8 | A | G | 0.5118 | Mood swings |
| rs34656207 | TBC1D22B | 6 | T | C | 0.3685 | Rheumatoid arthritis, Ankylosing spondylitis, Diabetes, Disability or infirmity |
| rs9296413 | CRIP3 | 6 | T | C | 0.6110 | Hypertension |

SNP, single nucleotide polymorphism; Chr, chromosome; EA, effect allele; OA, other allele; EAF, frequency of effect allele.

**Table S3.** The ‘‘leave-one-out” sensitivity analysis showed that the result was not affect by a single SNP.

| **SNP** | **b** | **se** | ***P*-value** |
| --- | --- | --- | --- |
| rs10901863 | 0.112 | 0.040 | 0.005 |
| rs11238325 | 0.103 | 0.040 | 0.008 |
| rs11881070 | 0.108 | 0.039 | 0.006 |
| rs13147559 | 0.101 | 0.040 | 0.010 |
| rs13172686 | 0.131 | 0.040 | 0.001 |
| rs1566129 | 0.109 | 0.039 | 0.005 |
| rs36062310 | 0.089 | 0.041 | 0.029 |
| rs4732339 | 0.106 | 0.039 | 0.006 |
| rs4859223 | 0.106 | 0.039 | 0.006 |
| rs55635402 | 0.099 | 0.039 | 0.011 |
| rs5756799 | 0.107 | 0.039 | 0.007 |
| rs67307131 | 0.010 | 0.040 | 0.012 |
| rs6902016 | 0.099 | 0.040 | 0.012 |
| rs72930982 | 0.096 | 0.039 | 0.014 |
| rs741475 | 0.094 | 0.039 | 0.016 |
| rs7525101 | 0.094 | 0.039 | 0.016 |
| rs78417468 | 0.094 | 0.039 | 0.016 |
| rs9493627 | 0.093 | 0.039 | 0.019 |
| All | 0.102 | 0.038 | 0.007 |

**Table S4.** MR analyses of association between HI and falls that used all extracted SNPs.

| **Methods** | **OR** | **LCI** | **UCL** | ***P*-value** |
| --- | --- | --- | --- | --- |
| Fixed-effect IVW | 1.130 | 1.041 | 1.227 | 0.004 |
| Weighted median | 1.156 | 1.051 | 1.272 | 0.003 |
| Simple mode | 1.165 | 0.952 | 1.426 | 0.151 |
| Weighted mode | 1.175 | 0.967 | 1.429 | 0.120 |
| MR-Egger | 1.041 | 0.779 | 1.391 | 0.790 |

*P<0.05, **P<0.01, ***P<0.001

*MR*, Mendelian randomization; *HI*, hearing impairment; *SNP*, single nucleotide polymorphism; *OR*, odds ratio; *LCI*, lower confidence interval; *UCI*, upper confidence interval; *IVW*, inverse variance weighted.

**Supplemental figure**


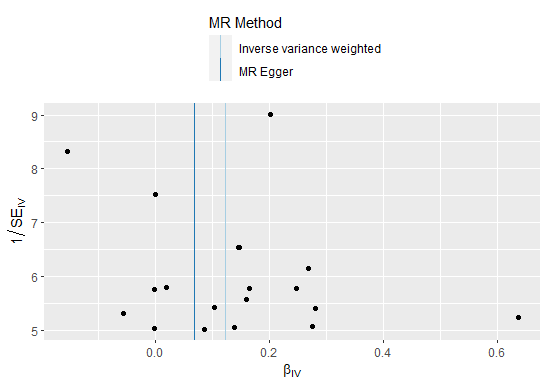
**Figure S1.** Funnel plot of the MR analysis for causal association of HI with falls. X axis presented the causal estimates and Y axis presented the inverse SE. The dots indicated each SNP, and the line indicated the overall estimate using fixed-effect IVW method. *MR*, Mendelian randomization; *HI*, hearing impairment; *SE*, standard error; *SNP*, single nucleotide polymorphism; *IVW*, inverse variance-weighted.
